# An observational study of the reactogenicity and immunogenicity of 13-valent pneumococcal conjugate vaccine in women of childbearing age in Papua New Guinea

**DOI:** 10.1186/s41479-020-00076-1

**Published:** 2020-11-25

**Authors:** Sarah Javati, Geraldine Masiria, Arthur Elizah, John-Paul Matlam, Rebecca Ford, Peter C. Richmond, Deborah Lehmann, William S. Pomat, Anita H. J. van den Biggelaar

**Affiliations:** 1 Papua New Guinea Institute of Medical Research, Homate Street, Goroka, Eastern Highlands Province 441 Papua New Guinea; 2 Division of Pediatrics, School of Medicine, Perth Children’s Hospital, University of Western Australia, 15 Hospital Avenue, Nedlands, WA 6009 Australia; 3 Wesfarmers Centre of Vaccines and Infectious Diseases, Telethon Kids Institute and Centre for Child Health Research, University of Western Australia, 15 Hospital Avenue, Nedlands, WA 6009 Australia

**Keywords:** Pneumococcal, Vaccine, Pneumococcal conjugate vaccine, PCV, Adults, Non-pregnant, Childbearing age, WOCBA, Safety, Immunogenicity, Papua New Guinea

## Abstract

**Background:**

Maternal immunization with pneumococcal conjugate vaccine (PCV) may protect young infants in high-risk settings against the high risk of pneumococcal infections in early life. The aim of this study was to determine the safety and immunogenicity of 13-valent PCV (PCV13) in healthy women of childbearing age in PNG.

**Methods:**

As part of this observational study, 50 non-pregnant women of childbearing age (18-45 yrs. old) living in the highlands of PNG were vaccinated with a single dose of PCV13. Local and systemic reactogenicity were assessed 24–48 h after vaccination. Venous blood samples were collected before and 1 month after vaccination to measure PCV13 serotype-specific IgG antibody concentrations.

**Results:**

No severe adverse effects were reported during the 1-month follow-up period. IgG antibody concentrations significantly increased after vaccination for all PCV13 serotypes. One month after vaccination IgG antibody levels ≥2.5 μg/mL were reached in at least 75% of women for all PCV13 serotypes, except serotype 3, and ≥ 5 μg/mL in at least 75% of women for 7 serotypes (serotypes 6B, 9 V, 14, 18C, 19A, 19F and 23F).

**Conclusion:**

PCV13 is safe and immunogenic in women of childbearing age living in a high-risk setting in PNG. This supports the implementation of studies to investigate the safety and immunogenicity of maternal PCV vaccination in high-risk settings as a strategy to protect infants in these settings against the high risk of pneumococcal infections in early life.

**Trial registration:**

NCT04183322. Registered 3 December 2019 - Retrospectively registered

**Supplementary information:**

**Supplementary information** accompanies this paper at 10.1186/s41479-020-00076-1.

## Background

*Streptococcus pneumoniae* remains a leading cause of childhood disease and death [1]. Most cases occur in low-income countries in children under 12 months of age [1, 2]. Compared to children in low- or moderate-endemic settings, infants in high-risk settings become infected at a younger age, with disease incidence peaking before 6 months of age [2]. Nasopharyngeal colonization is an immediate precursor to disease [3, 4], and in high-risk settings infants become rapidly colonized and disease is likely to follow. In the highlands of Papua New Guinea (PNG), where this study was performed, 50% of infants are colonized with pneumococci by 19 days of age, and almost all will have carried pneumococci before they are 1 month old [5]. Early intervention is therefore key to reducing the high burden of disease.

With support from The Vaccine Alliance (Gavi), eligible low-resource countries - including PNG in 2014 - have introduced pneumococcal conjugate vaccines (PCV) into routine childhood immunization schedules. PCVs are effective in preventing vaccine serotype-associated invasive disease, pneumonia, and mortality in high-risk infants [6–9]. PCVs can also induce herd protection by reducing acquisition and carriage of vaccine serotypes, hence interrupting circulation of vaccine serotypes in the community [10]. The World Health Organization (WHO) recommends children in high-risk settings receive 3 doses of PCV, scheduled around 6, 10 and 14 weeks of age. Protective immunity, however, is not established until 2–4 weeks after completion of the primary schedule [11, 12]. In PNG where infants are given 13-valent PCV (PCV13) according to the national immunization schedule at 1, 2 and 3 months of age [13], infants therefore remain largely unprotected between birth and 4 months of age (or older when vaccination is delayed), which is also the age that the burden of pneumococcal infections peaks in high-risk settings.

One possible strategy to induce earlier protection is neonatal vaccination. Neonatal PCV vaccination studies have been conducted in PNG and Kenya, and have shown that this is a safe and feasible strategy [12, 14, 15]; however, a period of susceptibility remains between the time of completing all vaccination and when infants start producing a protective immune response. An alternative strategy, and maybe the only solution to protect infants in the first and most susceptible months of life, is maternal immunization.

Maternal immunization is based on the principle of inducing high titers of protective antibodies in pregnant women that are transferred in-utero from mother to child to protect newborns against infections until they have developed their own immunity [16]. Maternal immunization can also protect mothers from acquiring and transmitting the vaccine-related pathogen to their newborn infants. This is relevant for maternal pneumococcal vaccination in high-risk settings where pneumococcal acquisition and carriage persist into adulthood. We have earlier demonstrated that in PNG approximately 30% of pregnant women carry pneumococci at the time of delivery and that this is associated with increased risk for early pneumococcal carriage in the newborn [5]. Maternal PCV vaccination in these settings therefore has the potential of inducing early protection in newborns and reducing transmission risk of mother to child.

Maternal vaccination trials using pneumococcal polysaccharide vaccines (PPV) have been conducted in the past, including in PNG, but there is insufficient evidence that maternal PPV can reduce infant infections [17, 18]. The possible effect of maternal PCV vaccination in preventing infections in children in high-risk settings is not known. To our knowledge, only the findings of one maternal PCV immunization trial have been published, involving a phase I/II trial conducted in the United States (US) [19]. The potential of maternal PCV immunization to protect infants in high-risk settings against pneumococcal infections therefore remains unknown. Moreover, there are no data on the safety and immunogenicity of PCV in adults in high-endemicity settings, which may be different to that in adults in low-endemicity settings. Therefore, before embarking on an immunization trial involving pregnant women in PNG, we conducted an observational study of the safety and immunogenicity of PCV13 in healthy women of childbearing age (WOCBA) in PNG.

## Methods

### Study population

Fifty non-pregnant women of childbearing age (18-45 yrs. old) living in Goroka town or surrounding villages in the Eastern Highlands Province of PNG were recruited into the study. Goroka is the provincial capital (population ~ 25,000; altitude 1600 m) and the PNG Institute of Medical Research (PNGIMR) laboratories and clinic are adjacent to Eastern Highlands Provincial Hospital (EHPH), the only tertiary hospital in the area. Participants were mothers of former study children of our pneumococcal vaccine trials who were invited to participate in this study; or eligible PNGIMR and EHPH staff. The following exclusion criteria were applied: known hypersensitivity to any vaccine component; known/suspected to be immunocompromised (including known HIV, TB or leukemia); receipt of corticosteroids ≤30 days before recruitment; pregnant; not being well at the time of vaccination; and intention to migrate out of the study area in the month after vaccination.

### Ethical considerations

Written informed consent was obtained from all study participants before enrolment in the study. The study was reviewed and approved by the PNGIMR Institutional Review Board (IRB 1515) and the PNG Medical Research Advisory Committee (MRAC 16.13). This study was conducted according to Declaration of Helsinki International Conference Harmonisation Good Clinical Practice (ICH-GCP) and local ethical guidelines. This study is registered under (NCT04183322).

### Vaccination

All study participants received a single dose of PCV13 (Prevnar13®, Pfizer Inc.) administered intramuscular in the non-dominant upper arm. Each 0.5 mL dose of PCV13 contains *S. pneumoniae* capsular polysaccharides serotypes 1, 3, 4, 5, 6A, 7F, 9V, 14, 18C, 19A, 19F, 23F (2.2 µg) and 6B (4.4 μg), and non-toxic diphtheria CRM_197_ carrier protein (34 µg), adsorbed on 125 μg aluminium phosphate. PCV13 study doses were procured from Pfizer Australia and shipped under temperature-controlled conditions to PNG.

### Safety and reactogenicity assessments

Study participants were observed for 30 min after vaccination for any immediate reactions and seen again 24–48 h after vaccination to record expected and unexpected side effects. At the 1-month post-vaccination visit, participants were interviewed and asked whether they had been unwell since vaccination.

### Specimen collection

A venous blood sample (10 mL) was collected shortly before vaccination and 1 month after vaccination. Blood samples were processed at the PNGIMR laboratories within 2 h of collection. Serum aliquots were separated by centrifugation at 5000 x *g* for 10 min and stored at -20 °C.

### Immunogenicity assessment

Serum IgG antibodies against PCV13 serotypes, and non-PCV serotype 2 were measured using a WHO standardized pneumococcal enzyme-linked immunosorbent assay (ELISA) [20, 21] established earlier at PNGIMR using the human pneumococcal standard reference serum 007sp [22], 10 μg/mL cell wall polysaccharide (CPS), and 5 μg/mL of purified serotype 22F polysaccharide for pre-absorbance of samples to remove non-specific antibodies and increase the specificity of the assay [20].

### Statistical analysis

Based on levels of naturally acquired antibodies specific for PCV13-serotypes measured in PNG adults and cord samples in earlier studies [12, 17], it was calculated that with a sample size of 50, a power of 80% and a significance level of 0.05, at least a 2-fold rise in post-vaccination antibody responses could be detected for all vaccine serotypes.

Statistical analysis was performed using the statistical package SPSS (SPSS Inc.). IgG antibody levels were log-transformed into geometric mean concentrations (GMCs). The Wilcoxon paired samples test was used to test for statistical differences in antibody GMCs before and after vaccination. To test for statistical differences in the proportion of women with antibody titers above a specified threshold before and after vaccination, the non-parametric McNemar test for paired nominal data was used. A difference was considered significant at a *P* level of < 0.05.

## Results

### Population demographics and follow-up

A total of 50 healthy female volunteers of childbearing age were recruited and screened: all fulfilled the eligibility criteria and were vaccinated with PCV13 after enrolment into the study. The participants’ mean age was 30.9 years (95% confidence interval [CI] 28.8–33.0). Vaccine responses were assessed 1 month after vaccination for 48 women (96%): 2 women were lost to follow-up due to migration out of the study area.

### Safety and reactogenicity of PCV13

The most common side effect was pain at the injection site, which was reported by 50% of the participants, followed by headache (18%), drowsiness (18%), loss of appetite (18%), and limited arm movement (18%) (Table 1). None of the participants reported fever, muscle pain, fatigue, vomiting, or rash. No unexpected adverse events were reported, and there were no serious adverse events.

**Table 1:** Local and systemic side-effects 24–48 h after PCV13 vaccination

Local side effects	n/N	% (95% CI)
Pain	25/50	50% (36–64)
Redness	8/50	16% (6–26)
Swelling	3/50	7% (0–14)
Rash	0/50	0% (0–0)
Limited arm movement	9/50	18% (7–11)
**Systemic side effects**	**n/N**	**% (95% CI)**
Muscle pain	0/50	0% (0–0)
Headache	9/50	18% (7–11)
Fever (≥ 38 °C)	0/50	0% (0–0)
Chills	2/50	4% (0–9)
Drowsiness	9/50	18% (7–11)
Fatigue	0/50	0% (0–0)
Loss of appetite	9/50	18% (7–11)
Vomiting	0/50	0% (0–0)

### Immunogenicity

Vaccination with PCV13 resulted in a significant rise in serum PCV13 serotype-specific IgG antibodies 1 month after vaccination, confirming that PCV13 vaccination in non-pregnant adult women in PNG is immunogenic (Table 2, Additional File 1). The relative increase in serum IgG antibody concentrations differed between serotypes and was highest for serotype 23F (mean fold increase [MFI] 4.51) and lowest for serotypes 7F, 5 and 3 (MFI 1.34, 1.84, and 1.87, respectively) (Table 2). IgG antibody levels did not change for non-vaccine serotype 2: this confirms the observed increases in PCV13 serotype-specific antibody responses are vaccine-induced.

**Table 2:** Geometric mean concentrations and mean fold increases of serotype-specific IgG antibodies pre- and post-PCV13 vaccination

	GMC (μg/mL) (95% CI)		MFI (95% CI)
	pre-vaccination	post-vaccination	
**PCV13 serotypes**			
1	4.44 (3.57–5.51)	8.70 (7.09–10.66)	2.39 (1.89–2.89)
3	0.44 (0.35–0.56)	0.67 (0.50–0.90)	1.87 (1.49–2.25)
4	2.11 (1.65–2.69)	4.44 (3.53–5.58)	2.75 (2.07–3.43)
5	4.38 (3.56–5.38)	7.15 (5.96–8.59)	1.84 (1.54–2.13)
6A	3.49 (2.78–4.38)	7.14 (5.37–9.49)	2.71 (2.00–3.42)
6B	6.58 (5.25–8.26)	16.30 (12.71–20.92)	2.86 (2.26–3.36)
7F	5.08 (4.22–6.12)	6.49 (5.39–7.81)	1.34 (1.21–1.48)
9V	4.07 (3.30–5.02)	8.69 (7.04–10.72)	2.95 (2.46–3.43)
14	15.46 (12.25–19.51)	28.30 (22.83–35.09)	2.22 (1.76–2.68)
18C	3.30 (2.63–4.15)	9.42 (7.60–11.67)	3.82 (2.87–4.76)
19A	9.01 (7.48–10.85)	16.60 (13.13–20.98)	2.56 (1.68–3.43)
19F	9.24 (7.56–11.31)	17.62 (14.31–21.70)	2.53 (1.83–3.24)
23F	3.85 (3.05–4.86)	11.69 (9.47–14.43)	4.51 (3.27–5.75)
**Non-PCV13 serotype**			
2	2.96 (2.29–3.85)	3.09 (2.39–4.01)	1.17 (0.95–1.39)

Pneumococcal serotype-specific IgG antibody responses were assessed before and 1 month after vaccination with PCV13 in 48 non-pregnant women of childbearing age and geometric mean concentrations (GMCs) were calculated. For each serotype, the mean fold increase (MFI) in IgG concentrations weas calculated by taking the mean of the fold increase in IgG antibody response after vs. before vaccination for each individual participant.

### Proportion of women with IgG levels ≥2.5 μg/mL and ≥ 5 μg/mL

The serotype-specific antibody concentration that should be achieved in pregnant women to enable placental transfer of antibodies at a concentration high enough to protect newborns against serotype-specific pneumococcal infections for the first 3 up to 6 months of life is not known. Guided by our previous observations that in PNG infants pneumococcal serotype-specific IgG antibodies wane on average 4-fold between birth and 2 months of age [12] and that IgG concentrations ≥0.35 μg/mL are considered protective against invasive pneumococcal disease, we presumed that serotype-specific IgG concentrations ≥2.5 μg/mL and ≥ 5 μg/mL in vaccinated women might theoretically be sufficient to confer protection in newborns for the first 3 months and 6 months of life, respectively.

**Fig. 1:**
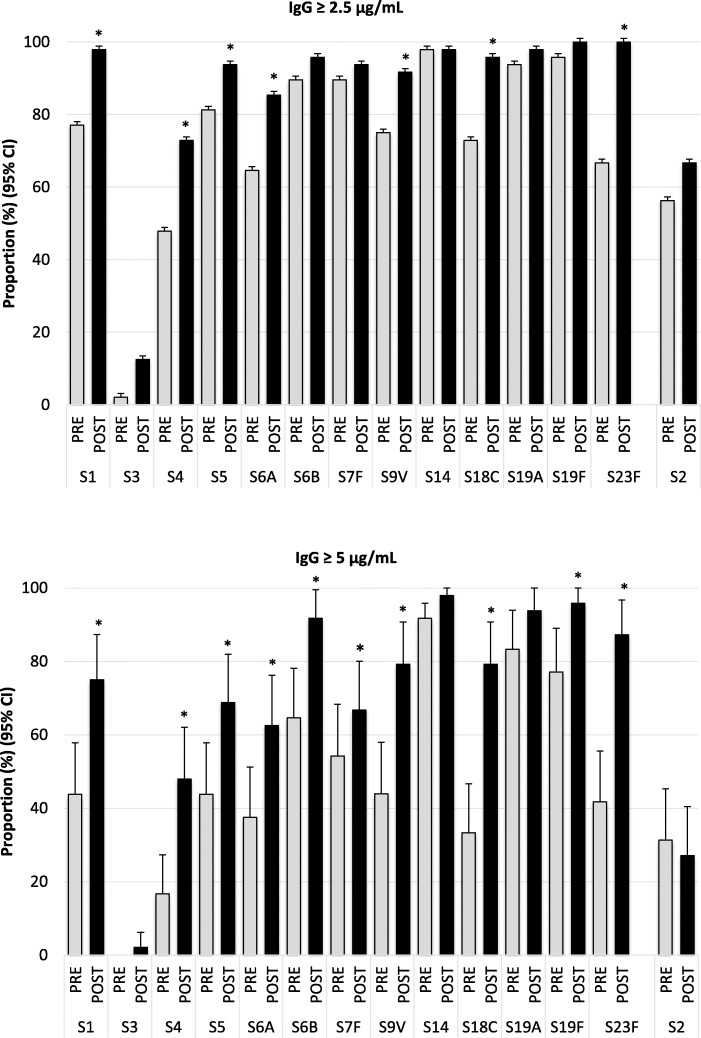
Proportions of women with serotype-specific IgG concentrations ≥2.5 or ≥ 5.0 μg/mL pre- and post-PCV13 vaccination. Serotype-specific IgG concentrations were measured before and 1 month after PCV13 vaccination. Thresholds of ≥2.5 μg/mL and ≥ 5.0 μg/mL were studied as indicators of antibody concentrations high enough to confer protection in newborns for the first 3 months and 6 months of life, respectively. Significant increases in the proportion of women with antibody titers above the specified threshold before and after vaccination are indicated with an asterisk (*, *p* < 0.05 based on McNemar test)

One month after PCV13 vaccination, IgG antibody concentrations ≥2.5 μg/mL were reached in at least 75% of women for all PCV13 serotypes, except for serotype 3 (Fig. 1). And concentrations ≥5 μg/mL were reached in at least 75% of women for 7 of the 13 serotypes (serotypes 6B, 9V, 14, 18C, 19A, 19F and 23F), whereas for serotypes 4, 5, 6A and 7F this concentration was reached in 48 to 69% of women (Fig. 1). For serotype 3 only 12.5 and 2% of women had IgG concentrations levels ≥2.5 μg/mL and ≥ 5 μg/mL, respectively.

## Discussion

To our knowledge this is the first study reporting on the safety and immunogenicity of PCV in non-pregnant women living in a setting where pneumococcal infections are highly endemic. Administration of PCV13 in healthy adult women of childbearing age in PNG was demonstrated to be safe and immunogenic. Comparable to reports for adults in low-endemicity settings [23], local site effects of PCV13 were mild and transient and there were no vaccine-related serious adverse effects. PCV13 vaccination resulted in increased serotype-specific IgG antibody titers in most vaccinated women 1 month after vaccination, confirming PCV13 is immunogenic in PNG adult women. As far as we are aware, only one other study has reported on PCV safety and immunogenicity in adults living in a high endemicity setting. Seventeen adults aged 18–40 years old were vaccinated with a candidate 10-valent PCV as part of a phase 1/2 trial in the Gambia but whether, or how many of these adults were males or females was not reported [24]. Local and systemic side effects were reported to be mild but data itself were not shown. Also pre-vaccination antibody titers were not presented; hence vaccine-induced antibody responses cannot be compared to our findings for PNG.

Immunogenicity varied between PCV13 serotypes. The greatest increase in IgG antibodies was found for serotype 23F, followed by serotype 18C. Serotype 7F was the least immunogenic, and responses were also relatively lower for serotypes 3 and 5. As expected for PNG adults who experience continuous high exposure and persistent nasopharyngeal pneumococcal colonization, pre-existing pneumococcal antibody levels were high. This is also reflected in the high levels of pneumococcal antibodies in newborns that we have reported on previously [5, 12]. There was no evidence to suggest that high pre-existing antibody levels influenced the relative immunogenicity of the different PCV13 serotypes. Serotype 3 is known to have limited efficacy and immunogenicity, probably because of its thick capsule [25]. We do not have an explanation for the limited immunogenicity of PCV13 serotypes 5 and 7F in the current study. In earlier studies involving PNG infants vaccinated with 10-valent PCV (PCV10) or PCV13, these serotypes were found to be immunogenic [26].

A limitation of our study is that we did not assess antibody functionality. In a study involving older adults in the US and Sweden, PCV13 was found to induce antibodies that were more opsonophagocytic than antibodies induced by PPV [27]. It is therefore possible that the antibodies induced by PCV13 are of a higher functionality or quality than the naturally acquired antibodies in these women. If that was the case, this would be relevant for the use of PCV13 as a maternal vaccine. After all, despite high levels of maternally-derived pneumococcal antibodies [12, 26, 28], PNG infants are at high risk of early pneumococcal colonization and disease. The functionality or protective capacity of maternal natural-acquired antibodies in newborns is therefore questionable. Moreover, maternal vaccination with PPV has been demonstrated to increase vaccine-specific antibody levels in maternal, cord and infant blood, and in breast milk [17, 29–31] but there is no evidence maternal PPV vaccination can protect infants against pneumococcal carriage, ear disease or respiratory illnesses [18, 32]. If it was demonstrated that antibodies induced by PCV have a higher capacity to prevent colonization or clear pneumococci than antibodies induced by PPV or natural infections, this would support the use of PCV as a maternal vaccine to protect high-risk young infants. In addition to measuring opsonophagocytosis or antibody avidity, it may be informative to assess antibody glycosylation patterns, as this may play a role in the selective transfer of antibodies across the placenta [33, 34]. Currently, the latter has not yet been established or studied for pneumococcal vaccines.

Another limitation is that we have no data on pneumococcal carriage in the participants to determine whether carriage has a serotype-specific effect on PCV immunogenicity in this population. We have previously shown that 30% of mothers of newborn infants living in the same setting carried pneumococci in the upper respiratory tract, with carriage rates being higher in younger than older mothers [5]. Serotyping was not performed in that study for the mothers; however, carriage studies conducted in PNG infants show that the spectrum of pneumococcal serotypes that is colonizing PNG infants is very broad even within the first month of life [35]. Further studies investigating pneumococcal carriage in PNG adults, including the spectrum, prevalence, and carriage load of different serotypes, and how this relates to risk of transmission to infants and vaccine responses in mother and child would be of great interest. This will require a relatively large population size as the prevalence of individual serotypes may be a limiting factor.

We believe that our findings that PCV13 is safe and immunogenic in non-pregnant adult women in PNG are sufficiently encouraging to pursue safety and immunogenicity studies with PCVs in pregnant women in PNG or other high-risk settings. Reactogenicity and immunogenicity may be different for pregnant and non-pregnant women, as well as that any potential effects on birth outcomes must be studied. The PROPEL trial that is taking place in The Gambia is investigating the immunogenicity of maternal PCV13 in 200 pregnant women; moreover, the study is comparing the effect of maternal PCV13 vaccination on pneumococcal colonization in infants to that in infants receiving a neonatal dose of PCV13 (born to non-vaccinated mothers) [36]. As many low-income countries have already introduced PCV into their childhood schedule, maternal PCV could be rapidly adopted without major practical or financial barriers by replacing 1 of the 3 infant doses with a maternal dose, if shown to be non-inferior.

## Conclusion

The demonstrated safety and immunogenicity of PCV in non-pregnant women in PNG supports as a next step studies investigating the safety and immunogenicity of PCV in pregnant women in high-risk settings. Maternal PCV vaccination, if shown to be effective, could be a strategy to protect infants in these settings against the high risk of pneumococcal infections in early life.

## Supplementary information

**Additional file 1 Supplementary Figure 1**. Serotype specific IgG responses before compared to after vaccination with one dose of PCV13 in 48 healthy women of childbearing age in Papua New Guinea. Serotypes included in PCV13 are depicted in blue; non-vaccine serotype 2 in red.

## Data Availability

The datasets generated and analysed during the current study are available from the corresponding author on reasonable request and when in accordance with ethical approvals.

## Abbreviations

CI: Confidence interval
EHPH: Eastern Highlands Provincial Hospital
ELISA: Enzyme-linked immunosorbent assay
Gavi: Global alliance for vaccines and immunization; the Vaccine Alliance
GMC: Geometric mean concentration
HIV: Human immunodeficiency virus
ICH-GCP: International conference on harmonisation good clinical practice
IgG: Immunoglobulin G
MFI: Mean fold increase
mL: Millilitre
PCV: Pneumococcal conjugate vaccine
PCV13: 13-valent pneumococcal conjugate vaccine
PNG: Papua New Guinea
PNGIMR: PNG Institute of Medical Research
PPV: Pneumococcal polysaccharide vaccine
TB: Tuberculosis
TKI: Telethon Kids Institute
US: United States
UWA: University of Western Australia
WHO: Word Health Organization
WOCBA: Women of childbearing age
