# Supplementary material for: An observational study of the reactogenicity and immunogenicity of 13-valent pneumococcal conjugate vaccine in women of childbearing age in Papua New Guinea
Source: Pneumonia (Nathan). 2020 Nov 25;12:13. doi: 10.1186/s41479-020-00076-1 (PMC7687988; doi:10.1186/s41479-020-00076-1)
Supplement: Supplementary file 1 — Additional file 1 Supplementary Figure 1. Serotype specific IgG responses before compared to after vaccination with one dose of PCV13 in 48 healthy women of childbearing age in Papua New Guinea. Serotypes included in PCV13 are depicted in blue; non-vaccine serotype 2 in red. [file 41479_2020_76_MOESM1_ESM.pptx]

## Slide 1
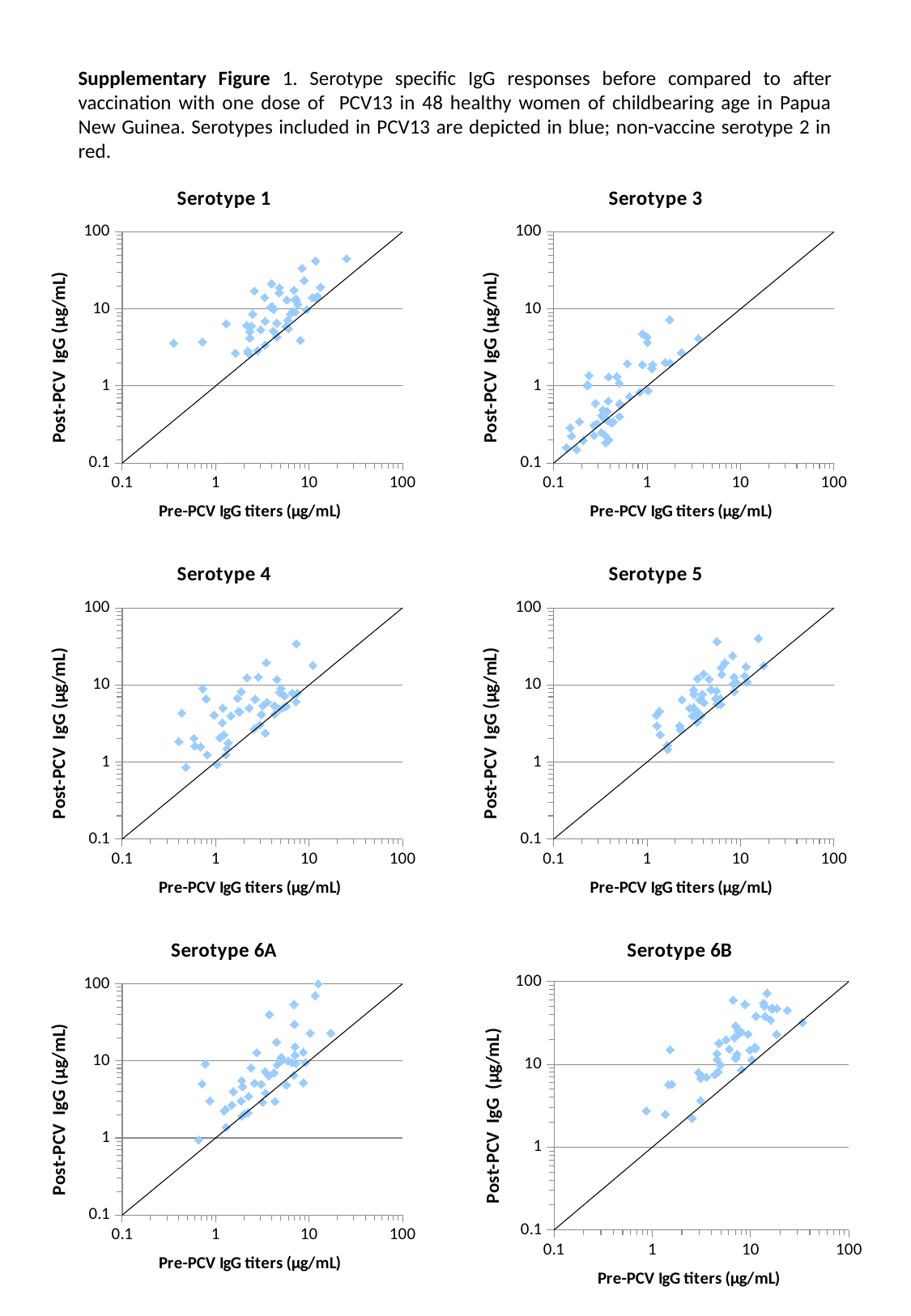

Supplementary Figure 1. Serotype specific IgG responses before compared to after vaccination with one dose of PCV13 in 48 healthy women of childbearing age in Papua New Guinea. Serotypes included in PCV13 are depicted in blue; non-vaccine serotype 2 in red.
### Chart: Serotype 1
| Category | S1_2 | Ref line |
|---|---|---|
### Chart: Serotype 3
| Category | S3_1 | Ref line |
|---|---|---|
### Chart: Serotype 4
| Category | S4_1 | Ref line |
|---|---|---|
### Chart: Serotype 5
| Category | S5_1 | Ref line |
|---|---|---|
### Chart: Serotype 6B
| Category | S6B_1 | Ref line |
|---|---|---|
### Chart: Serotype 6A
| Category | S6A_1 | Ref line |
|---|---|---|

## Slide 2
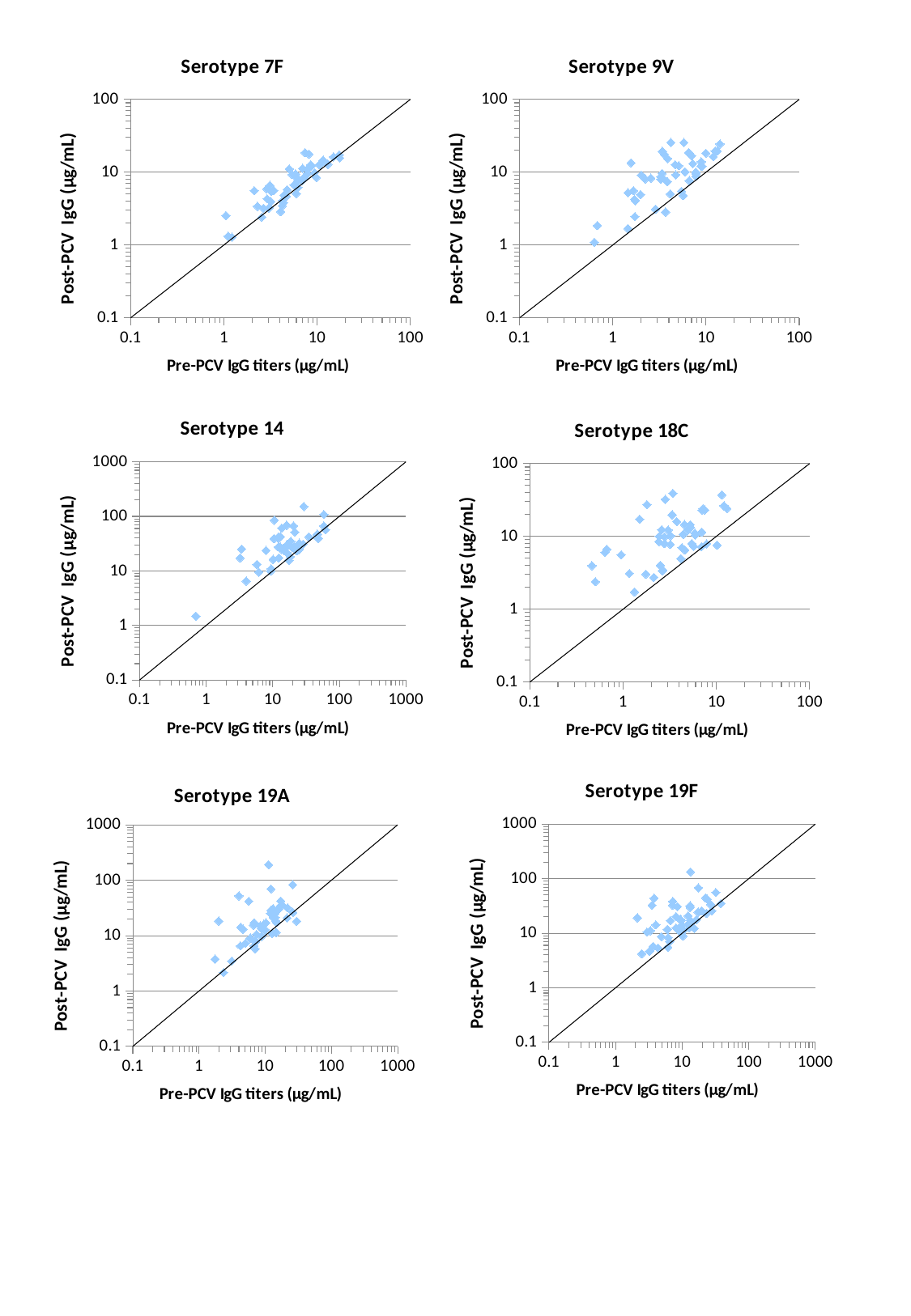

### Chart: Serotype 7F
| Category | S7F_1 | Ref line |
|---|---|---|
### Chart: Serotype 9V
| Category | S9V_1 | Ref line |
|---|---|---|
### Chart: Serotype 14
| Category | S14_1 | Ref line |
|---|---|---|
### Chart: Serotype 18C
| Category | S18C_1 | Ref line |
|---|---|---|
### Chart: Serotype 19F
| Category | S19F_1 | Ref line |
|---|---|---|
### Chart: Serotype 19A
| Category | S19A_1 | Ref line |
|---|---|---|

## Slide 3
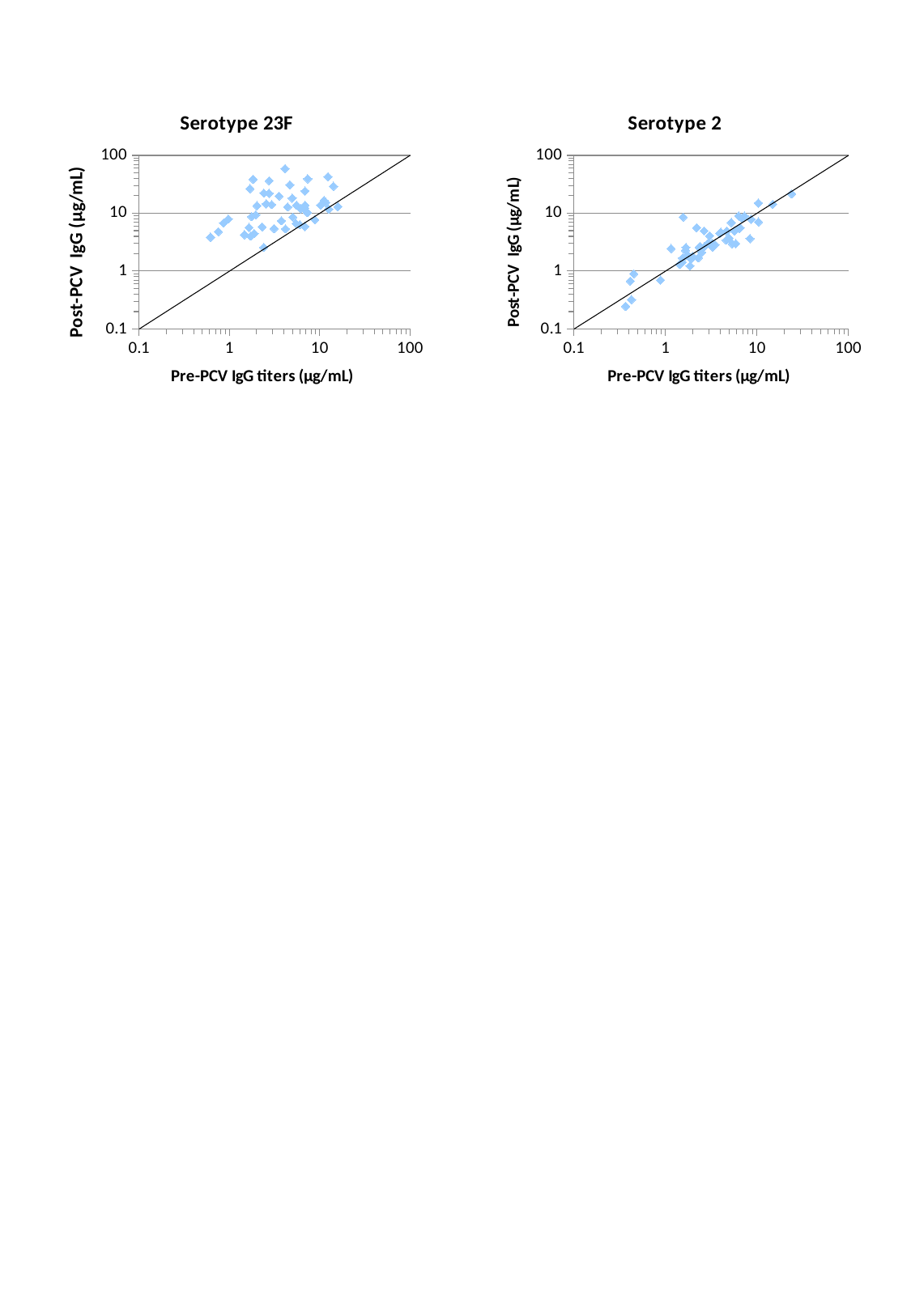

### Chart: Serotype 23F
| Category | S23F_1 | Ref line |
|---|---|---|
### Chart: Serotype 2
| Category | S2_1 | Ref line |
|---|---|---|
